# Commentary: Development and validation of cuproptosis-related gene signature in the prognostic prediction of liver cancer

**DOI:** 10.3389/fonc.2023.1159828

**Published:** 2023-07-03

**Authors:** Yi-Heng Du

**Affiliations:** ^1^ Research School of Biology, Australian National University, Canberra, ACT, Australia; ^2^ Shandong University and Australian National University (SDU-ANU) Joint Science College, Shandong University, Weihai, Shandong, China

**Keywords:** liver cancer, cuproptosis, lncRNA, mRNA, prognostic model

## Introduction

Liver cancer is a leading cause of cancer-related deaths worldwide. Early diagnosis and effective treatment options are crucial for improving the survival rate of liver cancer patients. In recent years, the application of next-generation sequencing technology has enabled the discovery of novel biomarkers for the diagnosis and prognosis of liver cancer. In this context, Liu et al. proposed a model for prognosis analysis of liver cancer patients based on cuproptosis-related gene expression. The model utilizes a combination of long noncoding RNAs (lncRNAs), tumor mutation burden (TMB), and drug prediction to predict the prognosis of liver cancer patients. However, as I have reviewed the paper, there are several concerns raised about the accuracy and validity of the results presented in the paper. These concerns include the specificity of the lncRNA selection, the contradictory TMB results, and the reliability of the drug prediction results. Therefore, further research and validation are needed to confirm the reliability and applicability of the model proposed in this paper.

## Discussion and conclusion

In Liu et al.’s paper (1), a model for prognosis analysis of liver cancer patients based on cuproptosis-related gene expression was proposed. However, upon reading the article, there are several questions. The first question is that in the determination of lncRNA, according to the results, 980 ferroptosis-related and not cuproptosis-related lncRNA were screened out, which may affect the accuracy of the model. This raises concerns about the specificity and robustness of the model. It is important to note that the selection of lncRNA is a key step in the construction of a prognosis model, and any inaccuracies in this step will inevitably lead to errors in the results.

The second question is that in the TMB results analysis, the original text mentions that the overall survival time of the high TMB group is higher than that of the low TMB group, but the results shown in Figure 6B (1) are exactly the opposite. This discrepancy raises questions about the validity of the conclusions drawn from the TMB analysis. Furthermore, the lack of a clear explanation for this discrepancy in the paper makes it difficult for readers to fully understand the implications of these results. Therefore, its conclusions are partially misleading.

The third question is about drug sensitivity. The R package used in this paper is pRRophetic (2), which compares its own dataset’s drug information, but it has not been updated since 2017, and the package’s corresponding author published a new package, oncoPredict (3), in 2021, which has similar functions as pRRophetic. This raises concerns about the reliability and applicability of the drug prediction results. With the rapid development of technology and new drugs being developed, it is important to use the latest tools and resources to ensure the most accurate predictions. Therefore, if oncoPredict is used for drug prediction, the results should be more accurate.

In summary, Liu et al.’s paper has partially misleading results; the lncRNA screened out is related to ferroptosis rather than cuproptosis, so the accuracy of the results should be considered. The analysis of TMB results is contradictory, which is confusing. In terms of tool usage, the package has not been updated for a long time, and the latest analysis tools were not used, so the predicted drugs may not be widely covered. Overall, the model proposed in this paper has potential, but further research and validation are needed to confirm its reliability and applicability. It is important to note that while the paper presents a unique model, it is not without its limitations and should be viewed with a critical eye. A deeper understanding of the underlying mechanisms and further validation are necessary before the model can be applied in the clinic.

## Author contributions

The author confirms being the sole contributor of this work and has approved it for publication.
